# Supplementary material for: Reaction-passivation mechanism driven materials separation for recycling of spent lithium-ion batteries
Source: Nat Commun. 2023 Aug 2;14:4648. doi: 10.1038/s41467-023-40369-9 (PMC10397256; doi:10.1038/s41467-023-40369-9)
Supplement: Supplementary file 1 — Supplementary Information [file 41467_2023_40369_MOESM1_ESM.pdf]

Supplementary Information

Reaction-passivation mechanism driven materials separation for recycling of spent lithium-ion batteries

Zihe Chen[a], Ruikang Feng[a], Wenyu Wang[a], Shuibin Tu[a], Yang Hu[a], Xiancheng Wang[a], Renming Zhan[a], Jiao Wang[a], Jianzhi Zhao[b], Shuyuan Liu[b], Lin Fu[a] and Yongming Sun[a, *]

[a] Wuhan National Laboratory for Optoelectronics, Huazhong University of Science and Technology, Wuhan 430074, China

E-mail address: yongmingsun@hust.edu.cn (Y. Sun).

[b] Mirattery Co., Ltd., Wuhan 430078, China

Supplementary Information include:

Supplementary Figures 1 to 44

Supplementary Tables 1 to 15

Supplementary Figures

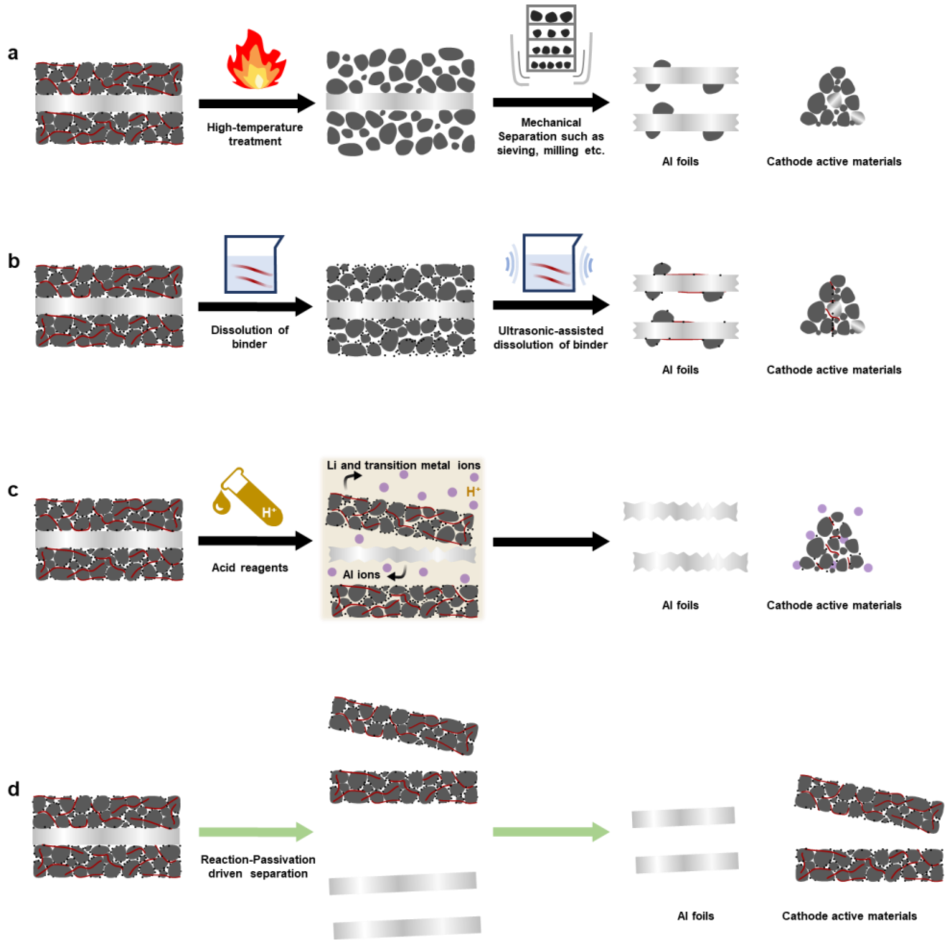

Figure S1. Schematics of strategies for Al foil-active material separation: (a) high-temperature treatment, (b) dissolution of binder in cathodes using organic solvents, (c) dissolution of Al foil with mineral acid and (d) reaction-passivation mechanism driven separation.

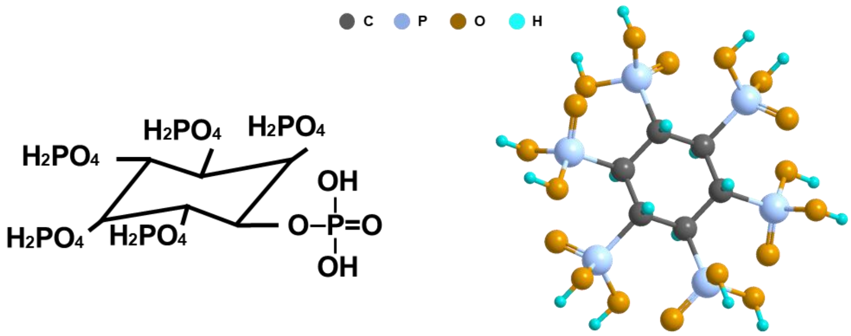

Figure S2. Structure of phytic acid (PA) molecular.

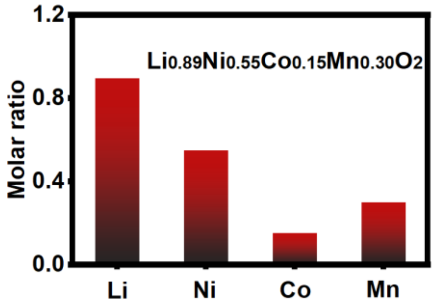

Figure S3. Molar ratios of Li, Ni, Co and Mn of spent cathode before separation according to the inductively coupled plasma mass spectrometry (ICP-MS) results.

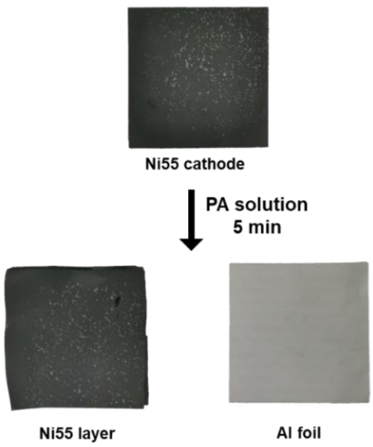

Figure S4. The digital images of a piece of $LiNi_{0.55}Co_{0.15}Mn_{0.30}O_2$ (Ni55) cathode before and after separation of Ni55 layer and Al foil.

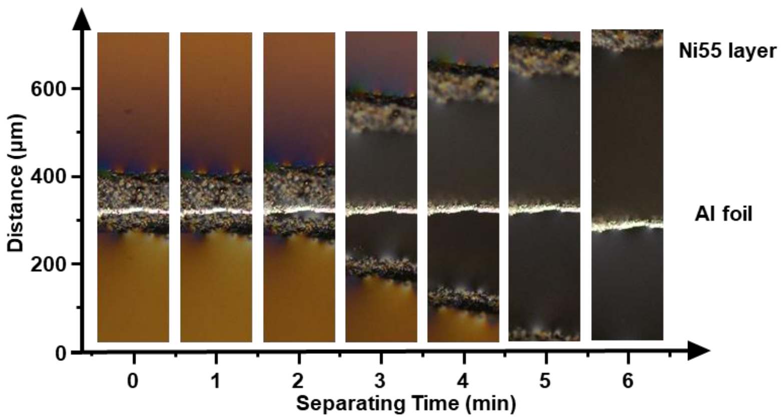

Figure S5. Cross-section optical microscope images of Ni55 cathode during its separation process in 30 wt% PA solution.

To reveal the effect of PA concentration on the separation time and Al passivation, we conducted the Al foil-Ni55 layer separation in PA solutions with concentrations of 10, 20, 30, 40 and 50% (namely 10 wt% PA-Al foil, 20 wt% PA-Al foil, 30 wt% PA-Al foil, 40 wt% PA-Al foil and 50 wt% PA-Al foil), respectively. The as-separated Al foils were investigated by potentiodynamic polarization and impedance spectroscopy. As showed in Figure S6-7 and Table S3, all of separated Al (S-Al) foils demonstrated similar small values of Ecorr, Icorr and Rct, indicating the similar good passivation effect of Al foil in the used PA solutions with different concentrations. Since 30 wt% PA solution enabled the shortest Al foil-Ni55 layer separation time (5 mins), and thus was investigated as the typical example (Figure S8).

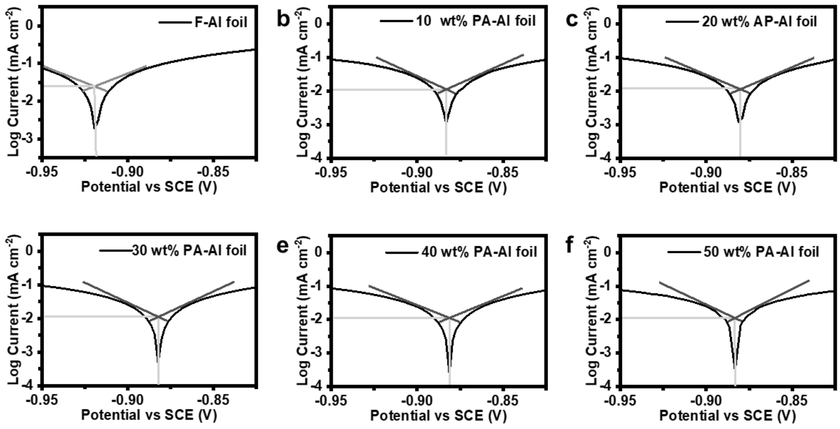

Figure S6. Tafel curves of (a) fresh Al (F-Al) foil and S-Al foils separated by using (b) 10, (c) 20, (d) 30, (e) 40 and (f) 50 wt% PA solutions, respectively.

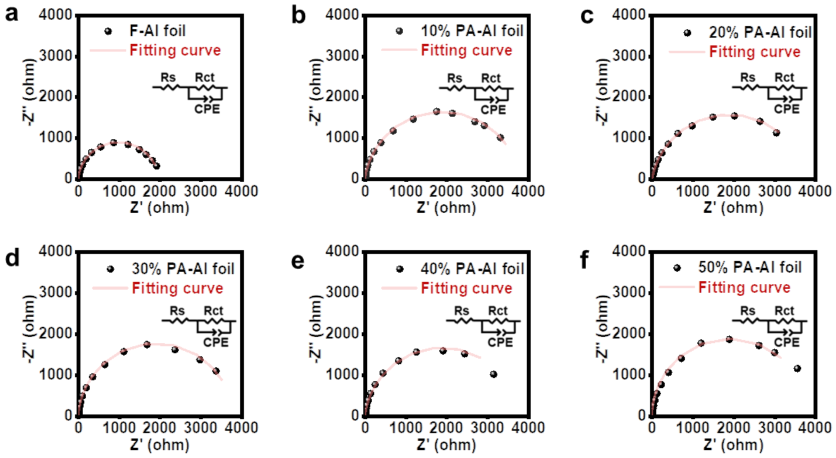

Figure S7. EIS plots of (a) F-Al foils and S-Al foils separated with (b) 10, (c) 20, (d) 30, (e) 40 and (f) 50 wt% PA solutions, respectively. The inset showed the equivalent circuit model.

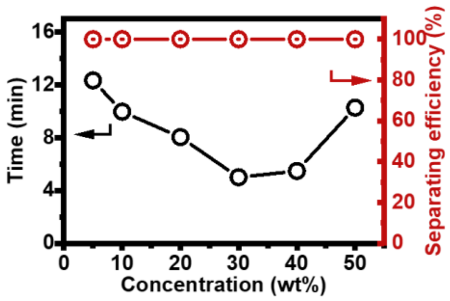

Figure S8. The rest time and separation efficiency dependence on PA solutions with different concentrations.

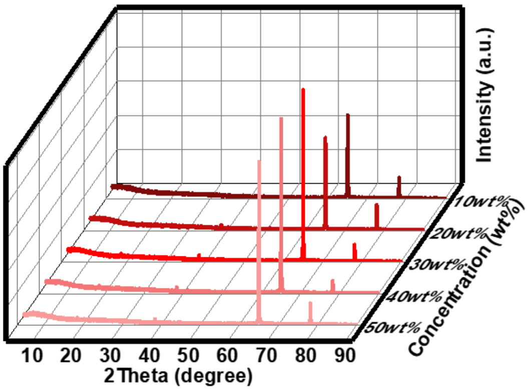

Figure S9. The XRD patterns of the S-Al foils using PA solutions with different concentrations.

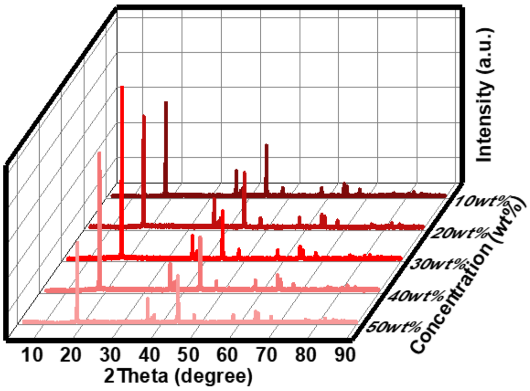

Figure S10. The XRD patterns of the separated Ni55 by PA solutions with different concentrations.

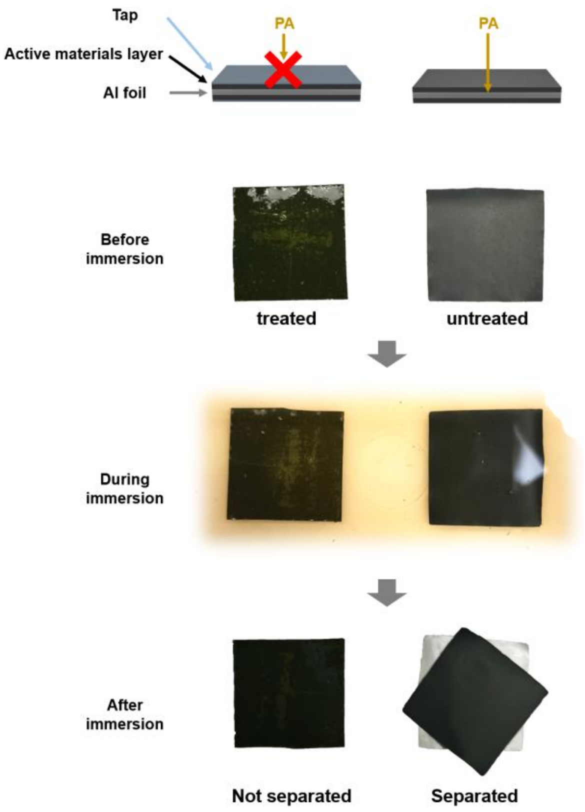

Figure S11. Schematic diagrams and digital images of the spent Ni55 cathode with covered tap for PA penetration behavior investigation. To reveal the reason for quick separation of Al foil and Ni55 layer, the top surface of a piece of Ni55 cathode was sealed, so that the PA solution could not penetrate from the top surface of the cathode, and only slight peeling of active material layer was shown on the edge of the cathode and the overall separation was hindered in sharp contrast to the fast peeling of the entire Ni55 layer from Al foil. These results indicated that the quick penetration of PA solution perpendicular to the electrode surface enabled the fast peeling of Ni55 layer from Al foil.

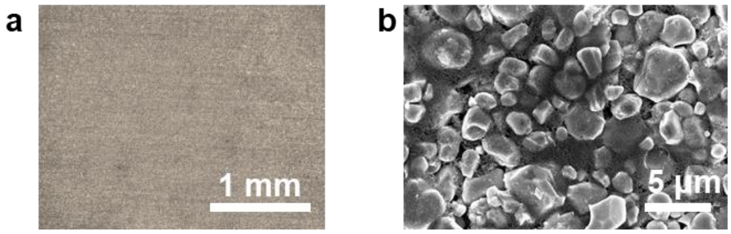

Figure S12. Optical microscope image of (a) S-Al foil and (b) Scanning electron microscope (SEM) image of Ni55 layer separated by using PA solution.

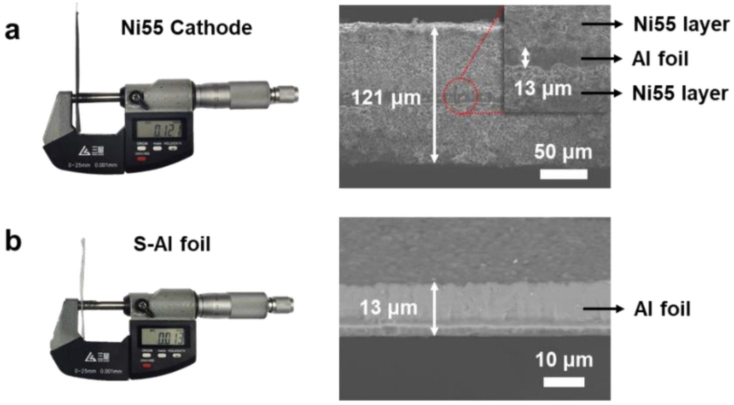

Figure S13. Digital images for thickness measurement by micrometer caliper and SEM images for (a) Ni55 cathode and (b) S-Al foil.

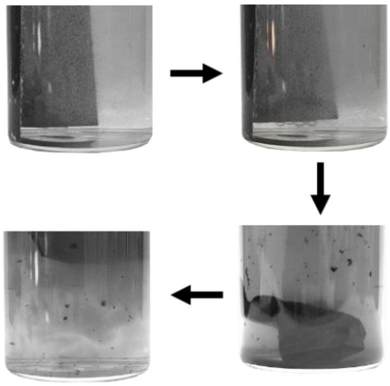

Figure S14. Digital images of a piece of Ni55 cathode during active material separation using HCl solution.

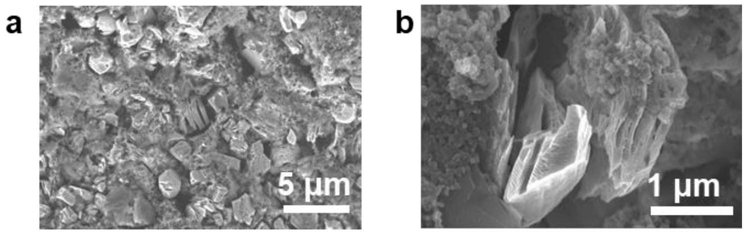

Figure S15. (a) Low and (b) high magnification SEM images of the Ni55 separated by using HCl solution.

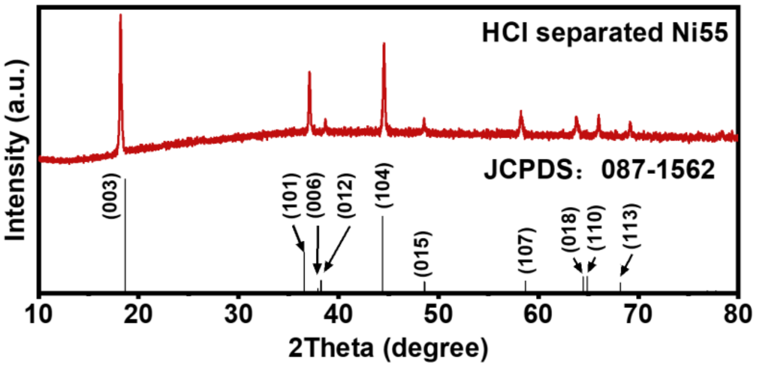

Figure S16. XRD patterns of the Ni55 separated by using HCl solution.

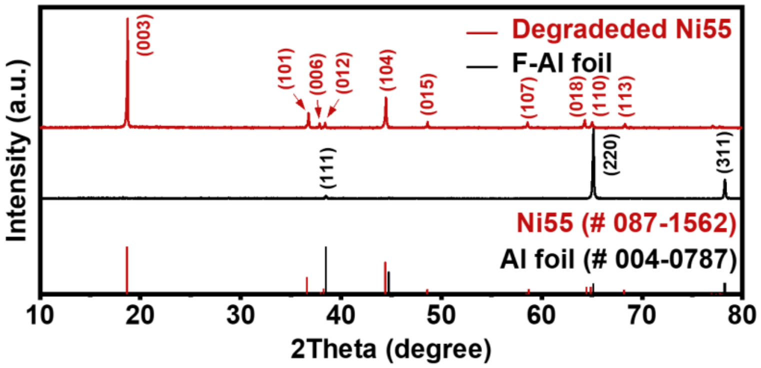

Figure S17. XRD patterns of F-Al foil and degraded Ni55.

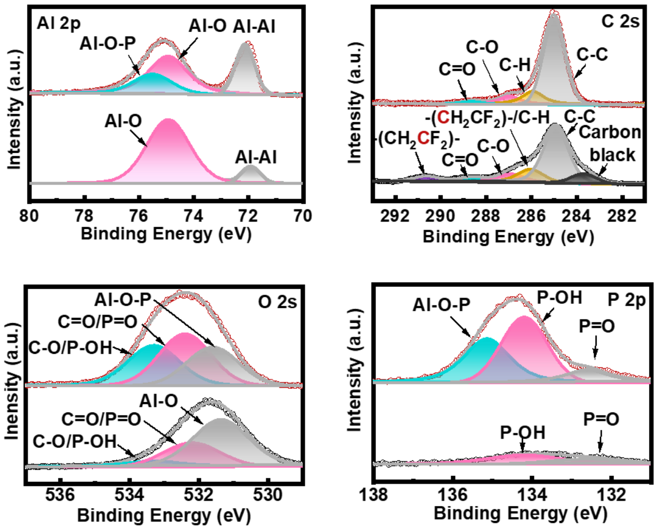

Figure S18. High-resolution Al 2*p*, C 2*s*, O 2*s* and P 2*p* X-ray photoelectron spectroscopy (XPS) spectra of Al foils separated by using (up) PA and (down) ultrasonic treatment in DI water.

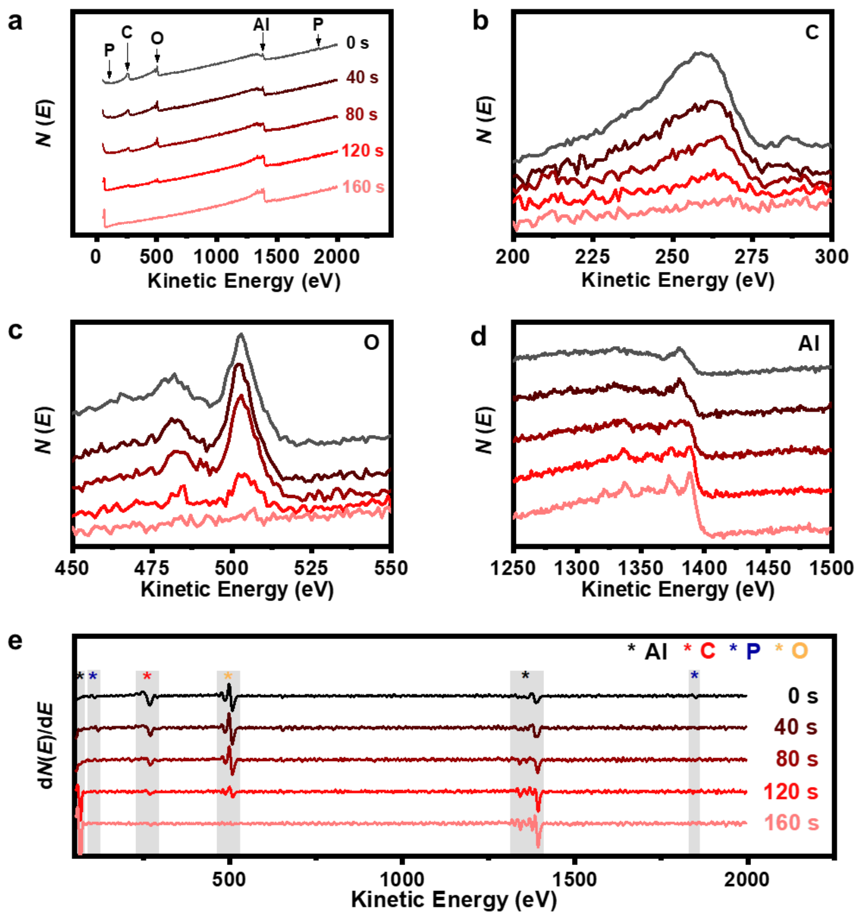

Figure S19. Auger electron spectra (AES) for aluminum-phytic acid complex (Al-PA) layer on Al foil with different Ar^+ sputtering times. (a) Direct spectra and (b-d) high-resolution spectra of (b) C, (c) O and (d)Al elements, and (e) the differential spectra of (a) for S-Al foil after different Ar^+ sputtering times. The existence of P, O and C elements was again verified, and all the signals were gradually reduced upon Ar^+ sputtering and disappeared after 160 s. Thickness of ~20 nm for the Al-PA layer was calculated based on silicon oxide wafer reference.

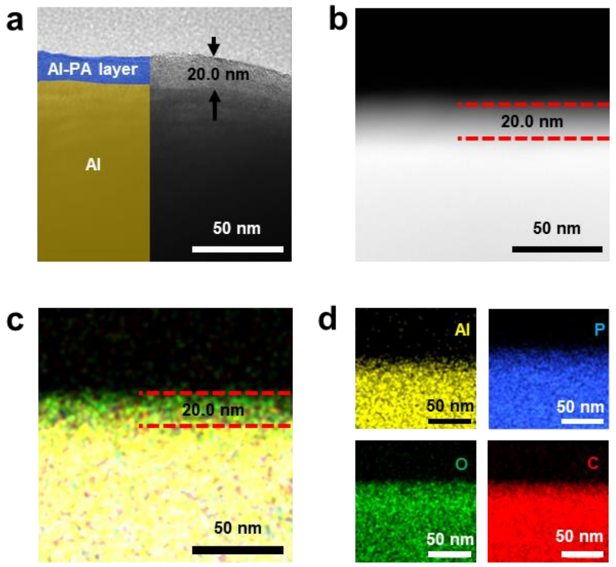

Figure S20. Transmission electron microscope (TEM) measurement for the Al-PA layer. (a) High-resolution TEM (HRTEM) image of Al-PA layer. (b) Dark-field TEM image and the corresponding (c) energy dispersive spectrometer (TEM-EDS) mapping images for (d) P, O, C and Al elements for Al-PA layer on Al. An amorphous surface layer of ~20 nm was observed on the surface of Al (Figure S20a-b). The existence of P, O, C and Al elements was confirmed by TEM-EDS mapping, which, together with the Fourier transform infrared spectroscopy (FTIR) results (Figure 3b), suggested the formation of dense Al-PA layer (Figure S20c-d). Correspondingly, the element content from the surface to inner was also plotted and ~20 nm in thickness was shown for the as-formed Al-PA layer.

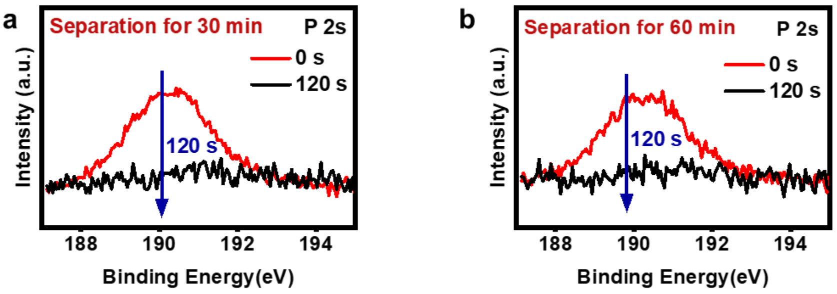

Figure S21. High-resolution P 2*s* XPS spectra of S-Al foils with PA treatment times of (a) 30 and (b) 60 mins. The signal for P-O peak in high-resolution P 2*s* spectrum disappeared after 120 s for both samples, consistent with the results for the S-Al foil with PA treatment time of 5 mins.

To show the generality of reaction-passivation mechanism driven Al foil-active material layer separation, we further performed the experiments on spent $LiCoO_2$ (LCO) cathodes. The results showed that the LCO layer was completely separated from the Al foil in PA solution in 5 mins. The obtained Al foil demonstrated a clean surface without any residual active material or observed damage (Figure S22). Besides, the thickness of the Al foil remained unchanged before and after separation (Figure S23). More elaborate measurement was further subject to ICP-MS measurement. The results indicated a low dissolution ratio (1.68 wt%) for LCO cathode in the used PA solution (Figure S24). The low Al content in the used PA solution and stable S-Al foil supported the formation of passivation layer on its surface. XRD measurement was further conducted to investigate the separated LCO materials. The separated LCO showed same XRD peaks as that before the PA treatment (Figure S25). Therefore, the above results verified that Al foil could be passivated instead of continuous corrosion after the initial reaction for Al foil-active material layer separation, where the similar results were also observed for spent $LiFePO_4$ (LFP) cathodes (Figure S26-27).

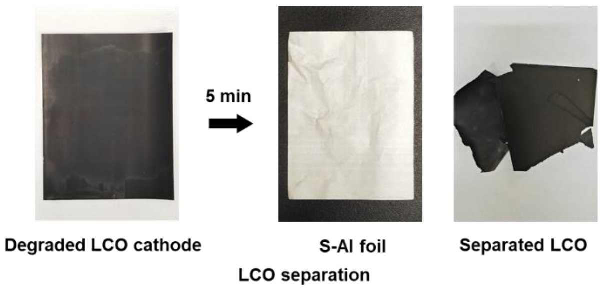

Figure S22. The digital images for Al foil and active material separation of LCO cathode in 30 wt% PA solution.

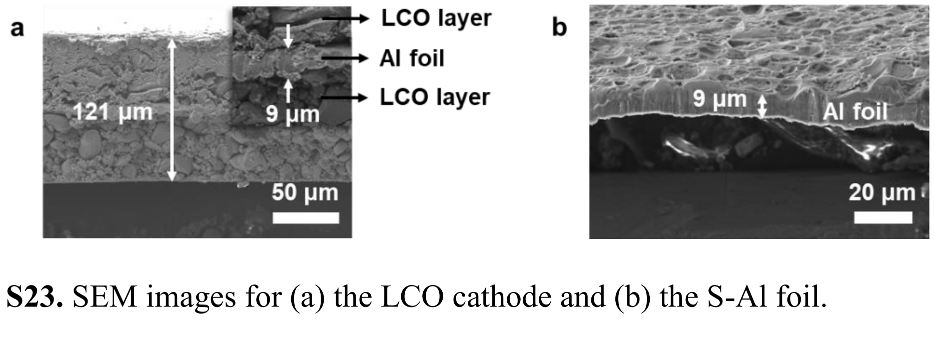

Figure S23. SEM images for (a) the LCO cathode and (b) the S-Al foil.

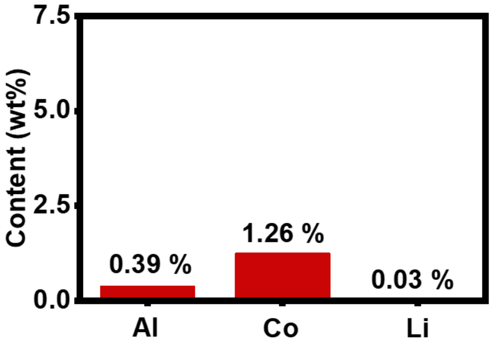

Figure S24. Al, Co and Li contents in PA solution after Al foil-LCO layer separation by ICP-MS measurement.

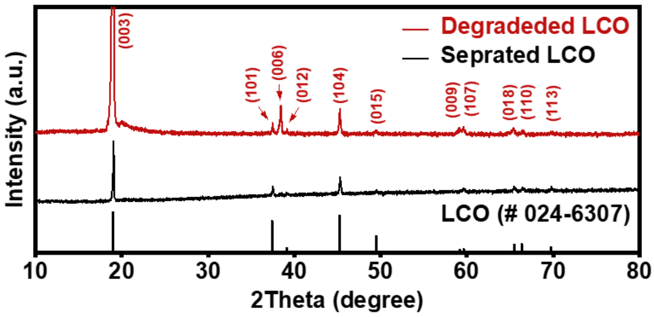

Figure S25. XRD patterns of the LCO before and after Al foil-LCO layer separation.

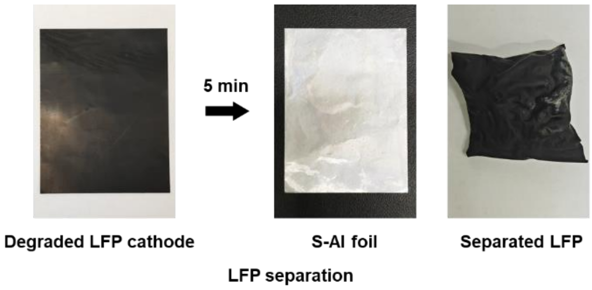

Figure S26. The digital images for Al foil and active material layer separation of LFP cathode in 30 wt% PA solution.

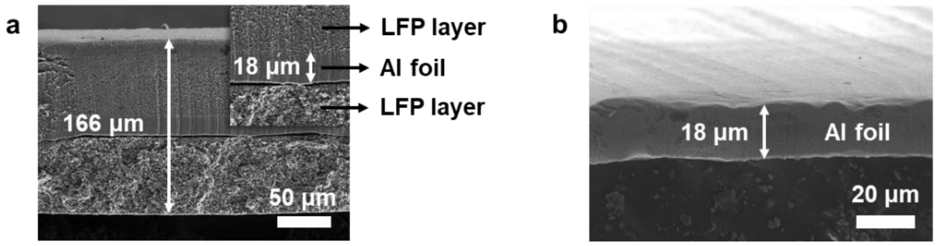

Figure S27. SEM images for (a) the LFP cathode and (b) the S-Al foil.

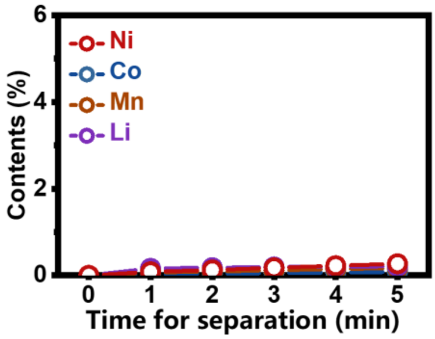

Figure S28. The dissolved Li, Ni, Co and Mn contents from Ni55 cathode in PA solution during Al foil-Ni55 layer separation.

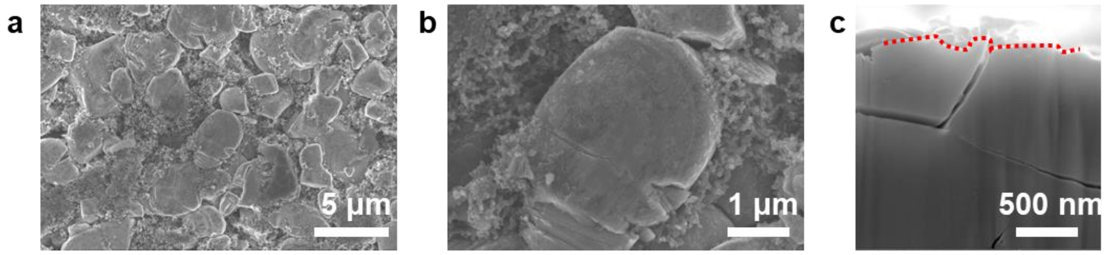

Figure S29. The (a-b) SEM and (c) focused ion beam electron microscopy (FIB-SEM) images of degraded Ni55 cathode.

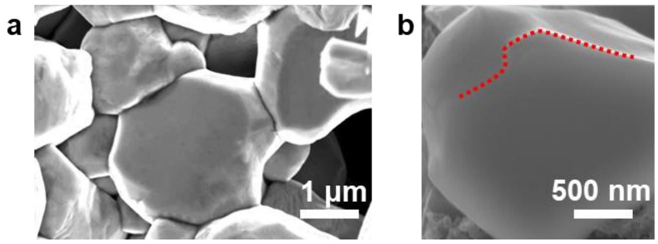

Figure S30. (a) SEM and (c) FIB-SEM images of regenerated Ni55.

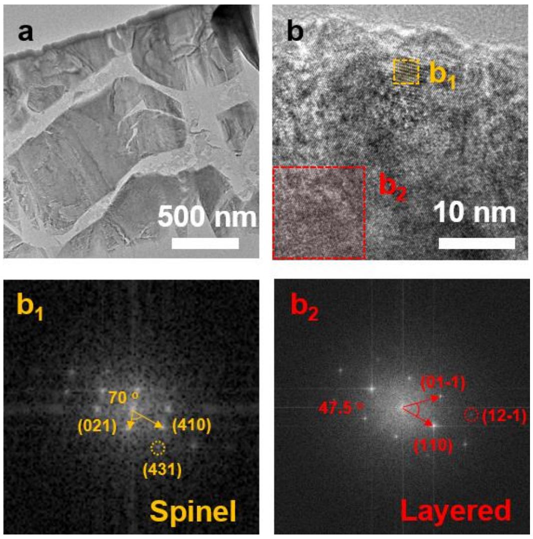

Figure S31. (a) FIB-TEM, (b) HRTEM images and (b1-b2) SAED patterns of degraded Ni55.

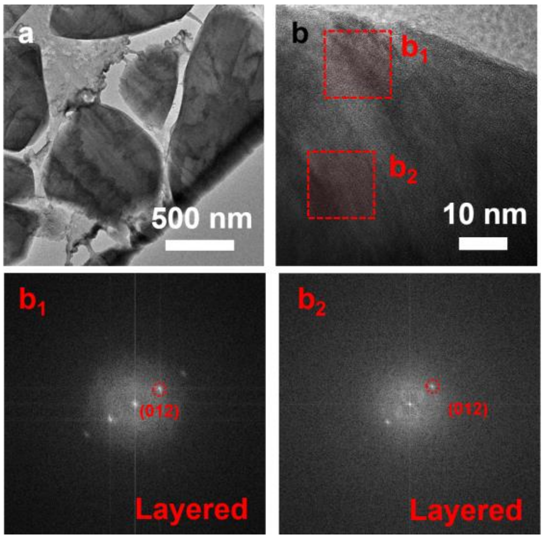

Figure S32. (a) FIB-TEM, (b) HRTEM images and (b1-b2) SAED patterns of regenerated Ni55.

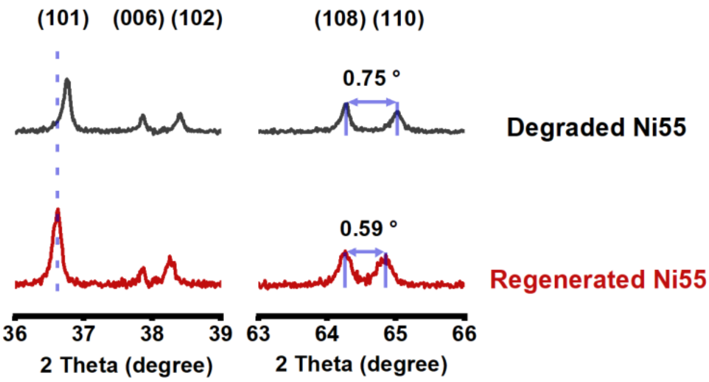

Figure S33. Zoom-in XRD peaks of degraded and regenerated Ni55.

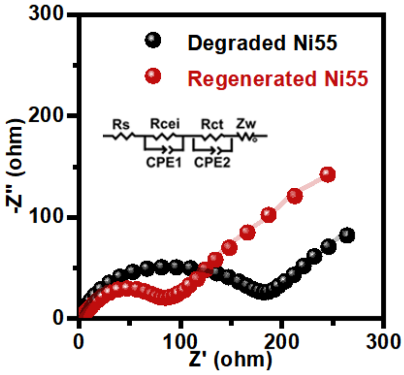

Figure S34. EIS plots of the degraded and regenerated Ni55. The regenerated Ni55 showed lower interfacial resistance and charge transfer resistance than the degraded counterpart, which supported the successful materials recovery. The inset showed the equivalent circuit model.

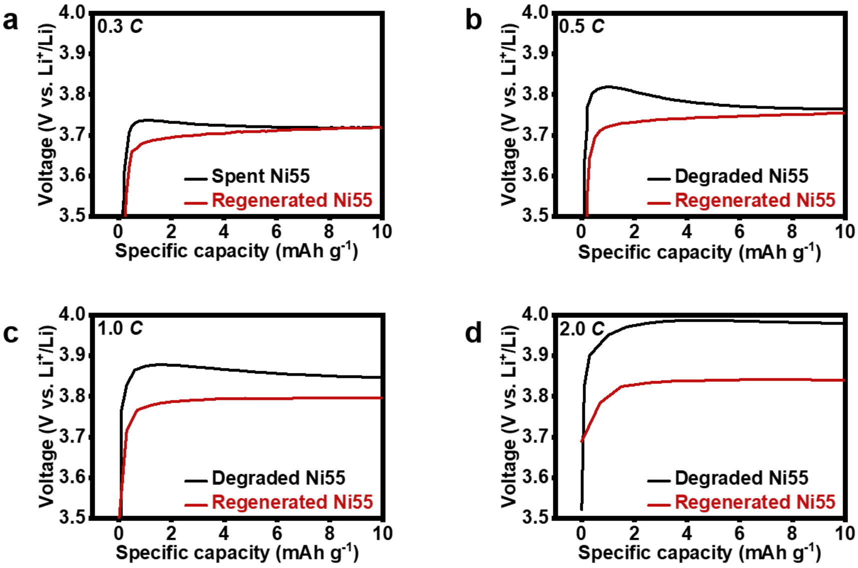

Figure S35. High-resolution galvanostatic charge curves for detailed overpotential difference in the initial stage of charging between the (black) degraded Ni55 and (red) regenerated Ni55 at (a) 0.3, (b) 0.5, (c) 1.0 and (d) 2.0 *C*. As shown in the voltage profiles, the overpotential of the regenerated Ni55 was reduced under different charge rates than the degraded Ni55, which indicated the improvement of electrochemical reaction kinetics for the regenerated Ni55.

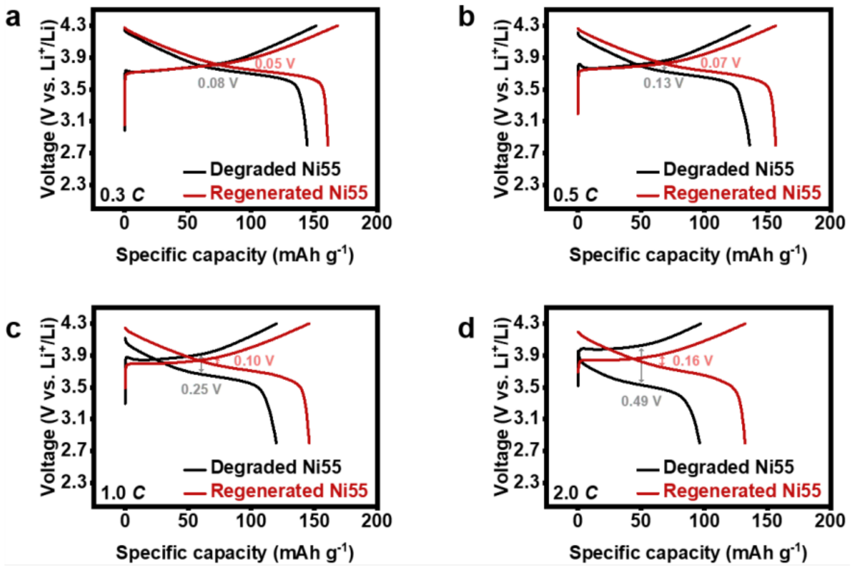

Figure S36. Galvanostatic discharge curve at (a) 0.3, (b) 0.5, (c) 1.0 and (d) 2.0 *C* of (black) degraded and (red) regenerated Ni55. After regeneration, the Ni55 displayed higher reversible capacities and lower potential hysteresis between charge and discharge curves at 0.3, 0.5, 1.0 and 2.0 *C* in comparison to the degraded Ni55, respectively.

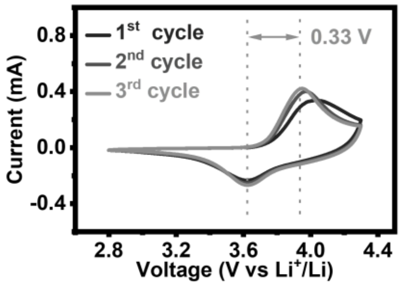

Figure S37. Cyclic voltammograms (CV) curves of degraded Ni55.

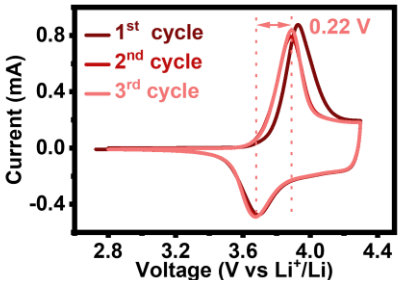

Figure S38. CV curves of regenerated Ni55.

The CVs of the degraded and regenerated Ni55 demonstrated voltage differences of 0.33 and 0.22 V for the redox couples, respectively, which suggested enhanced electrochemical reaction kinetics of the Ni55 after regeneration (Figures S37 and S38).

Economic and environmental analysis

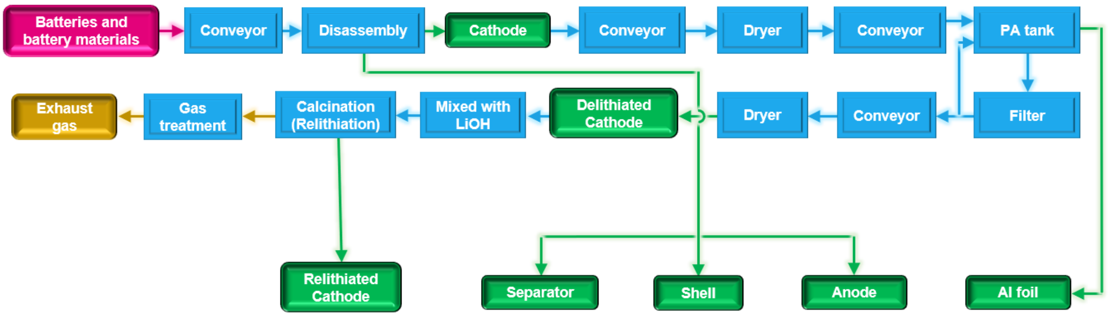

Figure S39. Process diagram of PA involved direct recycling (PA-direct).

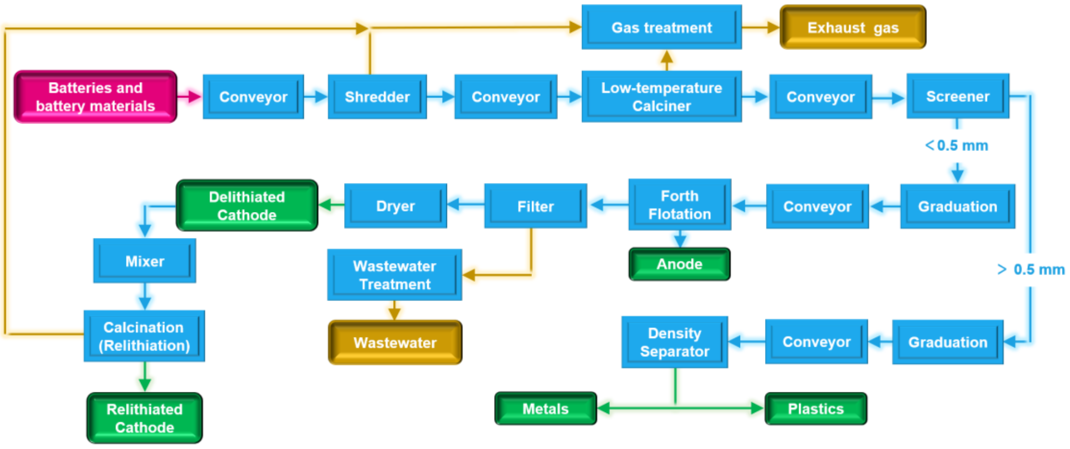

Figure S40. Process diagram of general direct recycling (General-direct).

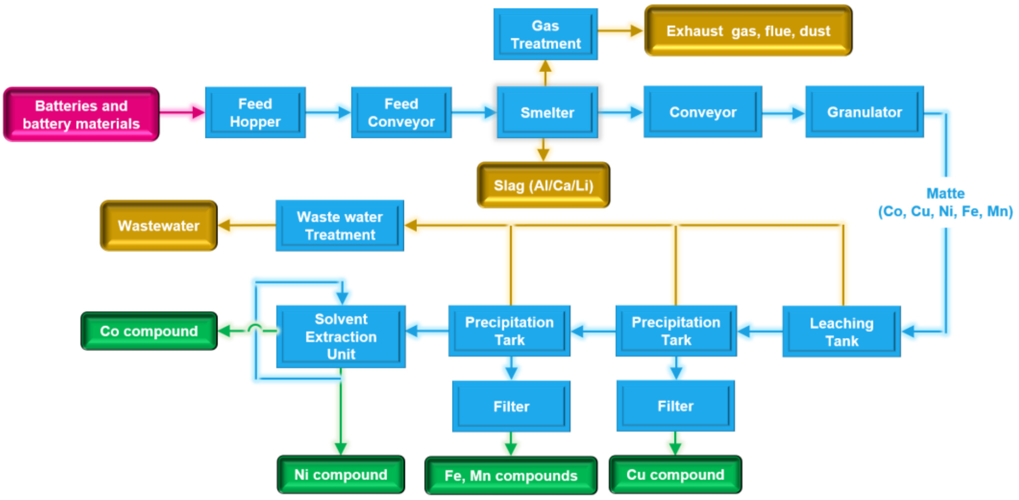

Figure S41. Process diagram of general pyrometallurgical recycling (Pyro).

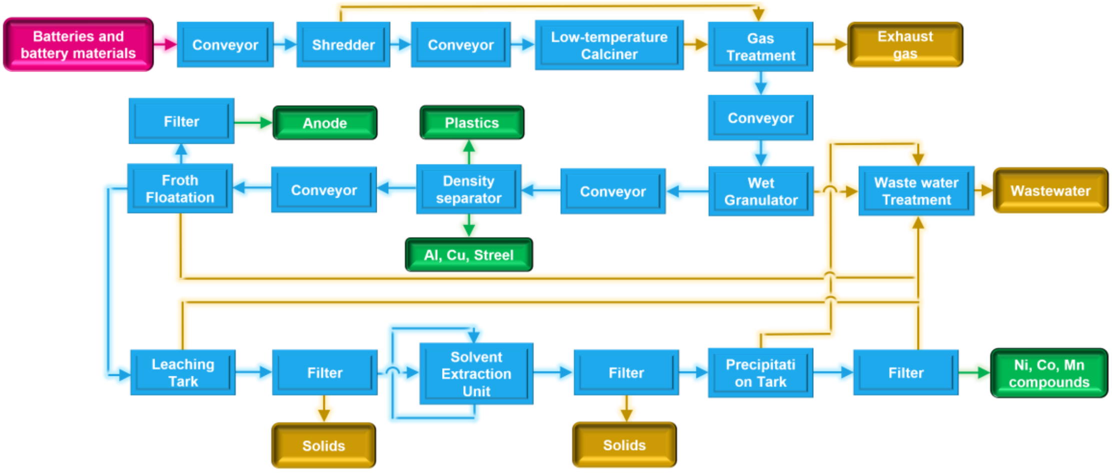

Figure S42. Process diagram of general hydrometallurgical recycling (Hydro).

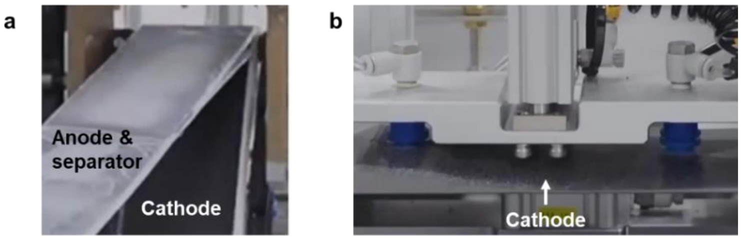

Figure S43. Digital images for (a) automatic disassembly of 102 Ah-spent Ni55 cell and (b) the obtained cathode.

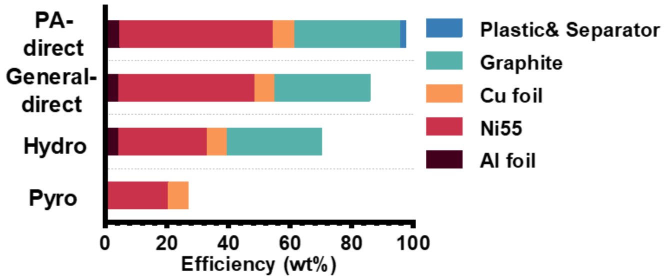

Figure S44. Recycling efficiencies of different recycling approaches.

Supplementary Tables

Table S1. ICP-MS results of the regenerated and degraded Ni55 before and after separation using 30 wt% PA and 30 wt% HCl solutions.

Types	Al (wt%)	Al / Li / Ni / Co / Mn (atomic ratio)
Degraded Ni55	0.016	0.063 / 89.457 / 54.955 / 15.131 / 29.914
Separated by using 30 wt% PA	0.026	0.100 / 89.478 / 55.146 / 14.989 / 29.866
Separated by using 30 wt% HCl	0.053	0.212 / 59.270 / 55.362 / 14.878 / 29.760
Regenerated Ni55	0.030	0.124 / 105.928 / 55.204 / 14.964 / 29.832

Table S2. Separation time, separation efficiency and elemental compositions (according to the ICP-MS test) of the obtained Al foils separated by using different reaction solutions.

Separation solution	Time (min)	Al / Li / Ni / Co / Mn (weight ratio)	Efficiency (wt%)
5 wt% PA	12.3	99.965 / 0.004 / 0.022 / 0.001 / 0.008	99.96
10 wt% PA	10.0	99.963 / 0.006 / 0.022 / 0.001 / 0.008	99.96
20 wt% PA	8.0	99.964 / 0.005 / 0.022 / 0.002 / 0.008	99.96
30 wt% PA	5.0	99.965 / 0.004 / 0.021 / 0.002 / 0.008	99.96
40 wt% PA	5.5	99.961 / 0.006 / 0.022 / 0.003 / 0.008	99.95
50 wt% PA	10.3	99.956 / 0.006 / 0.025 / 0.001 / 0.011	99.95
30 wt% PA-2nd	4.9	99.962 / 0.006 / 0.021 / 0.001 / 0.009	99.96
30 wt% PA-3rd	5.0	99.964 / 0.004 / 0.022 / 0.002 / 0.008	99.96
30 wt% PA-4th	5.1	99.966 / 0.004 / 0.021 / 0.001 / 0.007	99.96
30 wt% PA-5th	5.1	99.963 / 0.003 / 0.021 / 0.003 / 0.008	99.96
HCl	5.0	None Al foil	None

Table S3. Equivalent circuit and the corresponding values of Rs, Rct, Rcei and Zw.

Types	Equivalent circuit	Rs(Ω)	Rct(Ω)	Rcei(Ω)	Zw(Ω)
F-Al foil		4.488	3801	-	-
10 wt% PA-Al foil		3.334	3782	-	-
20 wt%PA-Al foil	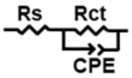	10.14	3766	-	-
30 wt% PA-Al foil (S-Al foil)		4.067	3793	-	-
40 wt% PA-Al foil		3.615	3762	-	-
50 wt% PA-Al foil		4.926	3798	-	-
Degraded Ni55	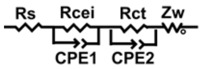	2.01	99.35	164.2	1862
Regenerated Ni55		1.712	62.16	105.4	368.9

Table S4. Oxidation peak (E_O), reduction peak (E_R) potential, and potential difference ($\Delta E = E_O - E_R$) of degraded and regenerated Ni55 cathode for the first, second and third cycles.

Sample	Degraded Ni55 cathode			Regenerated Ni55 cathode		
	1st	2nd	3rd	1st	2nd	3rd
E_O (V)	4.027	3.972	3.950	3.927	3.885	3.887
E_R (V)	3.621	3.621	3.624	3.691	3.681	3.675
ΔE (V)	0.406	0.351	0.326	0.236	0.204	0.212

Table S5. LCA and TEA of different recycling approaches.

	Pyro	Hydro	General-direct	PA-direct
Total energy (MJ kg^{-1}, cell)				
Total Energy	10.71	19.57	20.92	5.84
Fossil fuels	9.86	18.08	18.77	5.58
Coal	2.34	3.11	13.96	2.04
Natural gas	6.63	13.41	2.47	1.45
Petroleum	0.89	1.56	2.34	2.10
Water consumption (gal kg^{-1})	0.5	2.5	3.7	0.7
Total Emissions (g kg^{-1}, cell)				
VOC	0.12	0.21	0.32	0.21
CO	0.43	0.75	0.89	0.55
NOx	0.89	1.77	2.19	1.51
PM10	0.08	0.15	0.43	0.22
PM2.5	0.05	0.10	0.26	0.18
SOx	0.75	22.86	3.52	0.64
BC	0.02	0.02	0.05	0.05
OC	0.01	0.03	0.07	0.06
CH_4	1.18	2.12	2.54	0.73
N_2O	0.01	0.02	0.03	0.01
CO_2	2143	1396	1810	580
CO_2 (w/C in VOC & CO)	2144	1398	1813	582
GHGs	2183	1468	1896	605
Revenue ($ kg^{-1}, cell)	4.90	6.35	14.10	15.79
Cost ($ kg^{-1}, cell)	4.87	4.35	6.59	6.78
Profit ($ kg^{-1}, cell)	0.03	2.00	7.51	9.01

Table S6. Energy consumption of different recycling approaches (MJ kg^{-1}, cell).

	Material input	Energy input	Total
Pyro	7.72	2.99	10.71
Hydro	17.45	2.12	19.57
General-direct	4.79	16.12	20.92
PA-direct	4.17	1.67	5.84

Table S7. GHG emission of different recycling approaches (g kg^{-1}, cell).

	Material input	Energy input	Process
Pyro	0.48	0.22	1.48
Hydro	1.10	1.45	0.22
General-direct	0.48	1.35	0.07
PA-direct	0.41	0.13	0.07

Table S8. Water consumption of different recycling approaches (L kg^{-1}, cell).

	Material input	Energy input	Process
Pyro	1.29	0.68	0
Hydro	5.57	0.22	3.79
General-direct	2.49	7.90	3.79
PA-direct	2.14	0.44	0.07

Table S9. Materials requirements for 1 kg-spent cell recycling through different approaches.

Pyro	Hydro	General-direct	PA-direct
Hydrochloric acid	Sulfuric acid	Lithium hydroxide	Lithium hydroxide
Limestone	Hydrogen peroxide	Lime	PA solution
hydrogen peroxide	hydrochloric acid	Sodium hydroxide	
Sand	Soda ash		
	Sodium hydroxide		

Table S10. Prices for 50 wt% PA solution from different companies. The price information of PA solution (50 wt%) from 10 companies was collected in May 2023, and the average of which was ~5.76 \$ kg^{-1} for large-scale bulk purchase.

Website	Price (\$ kg^{-1})
http://www.scbczh.com/tf_product.asp?ln=0	5.81
http://gzdshg168.com/	5.81
http://61819185013.cn.gongxuku.com/credit/	8.34
https://wap.21food.cn/company/info1417749.html	5.81
http://www.jscwskj.com/	5.29
https://gzsanchanghg.company.lookchem.cn/	5.08
http://www.zzfthg.com/	7.55
https://sdacswkj.cn.china.cn/	3.78
http://www.condicechem.com/	3.63
http://www.hongtaobio.com/	6.54
Average price	5.76

Table S11. Prices of materials for different cathode recycling approaches.

	Price (\$ t^{-1})	Data sources
PA solution (50 wt%)	5764.20	Table S10
Ni55	32683.54	10jqka
$LiOH{\cdot}H_2O$	42561.23	SMM
Li_2CO_3	32756.17	SMM
graphite	4459.487	SMM
Al foil	2396.793	SMM
Cu foil	10110.11	SMM
HCl	8.71	100ppi
Ammonia	459.99	100ppi
Ammonium Bicarbonate	196.10	100ppi
Hydrogen Peroxide	115.24	100ppi
Ni in product	23355.19	SMM
Mn in product	2742.69	SMM
Co in product	30666.03	SMM
Water	1.75958248	BDB
Sewage treatment	2.20	51wctt

Data sources:

SMM (https://www.smm.cn/), 10jkqka (https://www.10jqka.com.cn/), 100ppi (https://www.101ppi.com/) and BDB (http://sz.bendibao.com/). The data was collected in May 2023.

Table S12. Cost for different recycling approaches ($ kg^{-1}, cell).

	Pyro	Hydro	General-direct	PA-direct
Materials	0.06	0.26	2.12	2.11
Labor	0.04	0.03	0.03	0.01
Other direct cost	0.30	0.17	0.22	0.26
Depreciation	0.48	0.26	0.31	0.38
Other fixed cost	0.54	0.30	0.35	0.42
Plant overhead	0.14	0.08	0.09	0.10
General expenses	0.24	0.17	0.42	0.44
Battery fee	3.05	3.05	3.05	3.05

Table S13. Produced materials from recycling approaches (kg kg^{-1}, cell).

	Pyro	Hydro	General-direct	PA-direct
Copper	0.05	0.05	0.05	0.06
Aluminum	NA	0.04	0.04	0.04
Graphite	NA	0.26	0.26	0.29
Mn in product	NA	0.07	NA	NA
Ni in product	0.13	0.13	NA	NA
Co in product	0.05	0.05	NA	NA
Ni55 product	NA	NA	0.38	0.42

Table S14. Revenue of different recycling approaches ($ kg^{-1}, cell).

	Pyro	Hydro	General-direct	PA-direct
Copper	0.55	0.55	0.55	0.61
Aluminum	NA	0.09	0.09	0.10
Graphite	NA	1.17	1.17	1.30
Mn in product	NA	0.19	NA	NA
Ni in product	3.20	1.15	NA	NA
Co in product	1.15	3.20	NA	NA
Ni55 product	NA	NA	12.30	13.79

Table S15. The TEA of manufacturing 1 kg-cell from raw and degraded Ni55 materials.

	Cost ($ kg^{-1})			Price of Ni55 ($ kg^{-1})	Profit ($ kg^{-1})
	Recycling	Regeneration	Production		
Virgin	NA	NA	26.41	32.68	6.27
Pyro	12.18	NA	12.14	32.68	8.36
Hydro	10.87	NA	7.86	32.68	13.95
General-direct	NA	17.52	NA	32.68	15.35
PA-direct	NA	16.07	NA	32.68	16.80
