# Supplementary material for: Reaction-passivation mechanism driven materials separation for recycling of spent lithium-ion batteries
Source: Nat Commun. 2023 Aug 2;14:4648. doi: 10.1038/s41467-023-40369-9 (PMC10397256; doi:10.1038/s41467-023-40369-9)
Supplement: Supplementary file 3 — Description of Additional Supplementary Files [file 41467_2023_40369_MOESM3_ESM.pdf]

Description of Additional Supplementary Files

Supplementary Movie 1: $LiNi_{0.55}Co_{0.15}Mn_{0.30}O_2$ layer was completely separated from the Al foil in 5 mins.
